# Optitrain: a randomised controlled exercise trial for women with breast cancer undergoing chemotherapy

**DOI:** 10.1186/s12885-017-3079-x

**Published:** 2017-02-06

**Authors:** Y. Wengström, K. A. Bolam, S. Mijwel, C. J. Sundberg, M. Backman, M. Browall, J. Norrbom, H. Rundqvist

**Affiliations:** 1 Department of Neurobiology, Care Science and Society, Division of Nursing, Karolinska Institutet, Karolinska University Hospital, 141 83 Huddinge, Stockholm, Sweden; 2 Department of Oncology, Karolinska University Hospital, Stockholm, Sweden; 3 Åstrand Laboratory of Work Physiology, The Swedish School of Sport and Health Sciences, Stockholm, Sweden; 4 School of Human Movement and Nutrition Sciences, The University of Queensland, Brisbane, Australia; 5 Department of Physiology and Pharmacology, Karolinska Institutet, Stockholm, Sweden; 6 Unit for Bioentrepreneurship, Karolinska Institutet, Solna, Sweden; 7 School of Health and Education, University of Skövde, Skövde, Sweden; 8 Department of Cell and Molecular Biology, Karolinska Institutet, Stockholm, Sweden

**Keywords:** Patients with breast cancer, Exercise intervention, Chemotherapy, Fatigue, Inflammation, Skeletal muscle

## Abstract

**Background:**

Women with breast cancer undergoing chemotherapy suffer from a range of detrimental disease and treatment related side-effects. Exercise has shown to be able to counter some of these side-effects and improve physical function as well as quality of life. The primary aim of the study is to investigate and compare the effects of two different exercise regimens on the primary outcome cancer-related fatigue and the secondary outcomes muscle strength, function and structure, cardiovascular fitness, systemic inflammation, skeletal muscle gene activity, health related quality of life, pain, disease and treatment-related symptoms in women with breast cancer receiving chemotherapy. The second aim is to examine if any effects are sustained 1, 2, and 5 years following the completion of the intervention and to monitor return to work, recurrence and survival. The third aim of the study is to examine the effect of attendance and adherence rates on the effects of the exercise programme.

**Methods:**

This study is a randomised controlled trial including 240 women with breast cancer receiving chemotherapy in Stockholm, Sweden. The participants are randomly allocated to either: group 1: Aerobic training, group 2: Combined resistance and aerobic training, or group 3: usual care (control group). During the 5-year follow-up period, participants in the exercise groups will receive a physical activity prescription. Measurements for endpoints will take place at baseline, after 16 weeks (end of intervention) as well as after 1, 2 and 5 years.

**Discussion:**

This randomised controlled trial will generate substantial information regarding the effects of different types of exercise on the health of patients with breast cancer undergoing chemotherapy. We expect that dissemination of the knowledge gained from this study will contribute to developing effective long term strategies to improve the physical and psychosocial health of breast cancer survivors.

**Trial registration:**

OptiTrain - Optimal Training Women with Breast Cancer (OptiTrain), NCT02522260; Registration: June 9, 2015, Last updated version Feb 29, 2016. Retrospectively registered.

## Background

It is well established that exercise has the potential to improve the health of cancer survivors; [1] however, evidence is still lacking on the benefits of different modalities of exercise and different exercise prescriptions on many common disease and treatment-related side-effects. Even less is known about the specific effects of different types of exercise on the most commonly reported side-effect, cancer-related fatigue [2–4]. Additionally, many existing exercise studies have been short in duration and lack follow-up assessments; thus, the long term-effects and sustainability of the exercise programme are not monitored or assessed. The few large randomised controlled exercise interventions in patients with breast cancer during chemotherapy have shown improvements in physical function, fatigue, and health-related quality of life (QoL) compared to patients receiving standard care [5, 6]. Differences have also been identified when comparing moderate or vigorous to a low intensity exercise program, demonstrating greater beneficial effects on QoL and physical function with moderate or vigorous exercise [7]. To develop exercise interventions and guidelines that have the potential to address the great problems facing cancer survivors, adequately-powered trials with long term follow-up are urgently required.

After a cancer diagnosis and during treatment, a marked deterioration of the individual’s physical capacity usually ensues and it is well known that patients with cancer generally reduce their activity levels after diagnosis [8]. Many patients are also forced to adapt their daily activities [9, 10] and side-effect symptoms resulting from treatment for cancer greatly affect the patients’ QoL. Previous research shows that QoL is a prognostic factor for patients with breast cancer that correlates with both survival and how well patients respond to treatment [11]. Although medications that target estrogen receptors decrease the risk for recurrence by approximately 50%, aromatase inhibitors result in detrimental musculoskeletal side-effects [12], possibly due to the influence of muscle protein turnover [13]. Other side-effects that patients with breast cancer encounter include cardiovascular complications, psychological distress [14] and increased levels of pain [15].

Despite growing research in oncology and physical exercise there is a paucity of randomised controlled trials that have particularly focused on investigating which modalities of exercise are most beneficial and at which intensity, duration and frequency these exercise modalities should be prescribed to reduce the side-effects and improve the health of patients with breast cancer [16]. While exercise trials for patients with cancer have generally employed either resistance or aerobic exercise, there is a lack of studies that have combined the two training modalities [1].

Significant barriers exist for patients with cancer who wish to return to work [17]. Exercise provides a potential strategy to improve the physical and mental health of survivors, in turn improving their likelihood to return to work, should they chose to. The PACES randomised clinical trial demonstrated that patients who were prescribed moderate to high intensity combined aerobic and resistance exercise or were prescribed light intensity physical activity during chemotherapy returned to work significantly earlier and for longer hours than the standard care group at the end of a 6-month intervention period [18]. As survival rates for breast cancer increase, facilitating successful return to work rates is of great importance.

Attendance and adherence to exercise protocols have generally been poorly monitored and reported in the existing literature, which may have resulted in an over- or underestimation of the effects of exercise programs. Investigation of the importance of adhering to exercise protocols and rates of attendance is warranted to understand the real- world implications of these exercise programs.

The molecular mechanisms induced by exercise in patients with cancer are still largely unknown. Low-grade systemic inflammation is linked to most events involved in the development and progression of cancer [19] and an ongoing elevated systemic inflammatory state is associated with poor prognosis in patients with cancer [20]. Findings on anti-inflammatory effects of physical exercise have mainly been demonstrated in healthy individuals showing that regular exercise exerts anti-inflammatory effects [21, 22]. Few studies have evaluated the effects of exercise on inflammatory markers after a cancer diagnosis and data on inflammatory responses to exercise during chemotherapy are even scarcer but tend to show exercise-induced anti-inflammatory effects [23, 24]. Those data are, however, confounded by heterogeneous patient populations, small sample sizes, and analysis of only a few pro/anti-inflammatory biomarkers [25]. Moreover, the effect of different types and doses of standardised exercise on inflammatory markers in patients with cancer need to be elucidated.

The current study will examine and compare the effects of different modalities and intensities of exercise on a wide range of clinically important and varying endpoints in patients with breast cancer. It will be the first study of its kind to offer supervised exercise sessions in the clinical setting that includes strategies to monitor physical activity levels over the course of and after the 16-week supervised intervention period including multiple follow up measurements (1, 2, and 5 years). In addition to functional outcomes, important biomarkers of systemic inflammation and skeletal muscle gene activity will be assessed. This can translate into better take out well-informed and targeted strategies for surveillance and treatment of patients with breast cancer.

The primary aim of the study is to compare the effects of two different exercise regimens (aerobic exercise vs. a combination of aerobic and resistance exercise) and a usual care control group on the primary outcome cancer related fatigue and the secondary outcomes cardiovascular fitness, muscle strength, function and structure, systemic inflammation, gene activity, health related quality of life, pain, disease and treatment related symptoms in patients with breast cancer receiving chemotherapy. The second aim is to examine if these effects, if any, are sustained one, two, and five years following the completion of the intervention and to monitor the effects of the intervention on return to work, recurrence and survival. The third aim of the study is to examine the effect of attendance and adherence rates on the effects of the exercise programme.

## Methods/design

### Study design

**Fig. 1:**
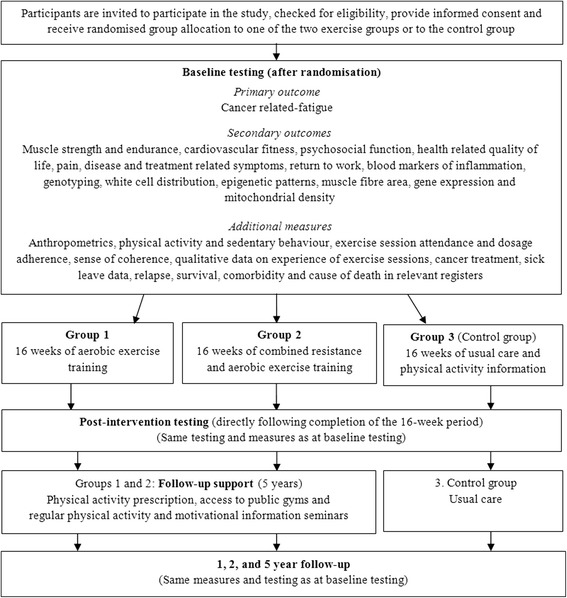
The modified SPIRIT study flow chart

This study is a 16-week randomised controlled exercise trial with follow-up at one, two and five years. The modified SPIRIT study flow chart is shown in Fig. 1. Briefly, the participants are randomly allocated to either: group 1: Aerobic exercise training (AT) twice a week for 16 weeks, group 2: Combined resistance and aerobic exercise training (CT) twice a week for 16 weeks, or group 3: usual care (UC) (control group) with physical activity information but no supervised training. To support the women to maintain exercising after the intervention during the 5-year follow-up period, participants in the exercise groups will receive a physical activity prescription, access to public gyms and invitations to regular motivational information seminars.

### Study setting

The study centre, testing and exercise clinic are located at Karolinska University Hospital, and Karolinska Institutet, Stockholm, Sweden.

### Ethics

Ethical permissions (with numbers 2012/1347-31/1, 2013/632-32 and 2014/408-32) have been obtained from the Regional Ethical Review Board in Stockholm. All participants will provide informed written consent prior to their entry into the study. In the case that changes in the protocol are necessary, relevant amendments will be made and submitted to the relevant ethics trial registration authorities.

### Participant recruitment

Recruitment began in March 2013 and is ongoing. Two-hundred and forty (80 in each group) women with breast cancer undergoing chemotherapy are being recruited from the Department of Oncology Breast and Sarcoma units at Radiumhemmet and Södersjukhuset, Karolinska University Hospital in Stockholm, Sweden. The expected completion date for recruitment is August 2016. Oncology physicians inform eligible patients about the study and, should they wish to participate, they fill in a cardiovascular health questionnaire and undergo an echocardiogram. If no relevant cardiovascular health issues are identified, the participants are deemed fully eligible for the trial. Once the participants have had any of their possible questions answered to their satisfaction and wish to volunteer for the study, the participants then sign the written informed consent form prior to data collection. Research nurses at The Clinical Trials Unit at Radiumhemmet then inform the study researchers of the participant’s eligibility and of the group allocation of the patient.

### Eligibility criteria

Inclusion criteria include women (aged 18–70 years) with Stage I-IIIa breast cancer about to start adjuvant chemotherapy. Exclusion criteria are assessed by study researchers and include patients with advanced disease, patients where physical activity is contraindicated because of safety reasons such as uncontrolled cardiovascular disease, brain or bone metastases, cognitive dysfunction, and patients who do not speak or understand the Swedish language.

### Randomisation process

The participants are randomly allocated by the Clinical Studies Unit at Radiumhemmet, Karolinska University Hospital (Stockholm, Sweden) to either AT, CT, or UC at a 1:1:1 ratio using a computer-generated program. A randomly selected subset of participants (*n* = 45) will also undergo a muscle biopsy before and after the intervention.

### Blinding

As this is an exercise intervention, participants cannot be blinded to group assignment. Outcome assessors are not blinded to group allocation, with the exception of assessors of muscle biopsy and blood samples who are blinded to group allocation.

### Testing

Data collection will occur prior to, directly after and at one, two and five years following the completion of the 16-week intervention period as shown in Fig. 1. The primary outcome is fatigue. Secondary outcomes are return to work, muscle strength and function, cardiovascular fitness, systemic inflammation, mitochondrial density, muscle fibre area, QoL, pain, disease status and treatment-related symptoms.

### Primary outcome

The Piper Fatigue Scale is being used to subjectively assess fatigue in four dimensions: behavioral/severity, affective meaning, sensory, and cognitive/mood [26]. The scale has been shown to have psychometric properties that are reliable for assessing subjective dimensions of fatigue among Swedish populations of patients with cancer [27].

### Secondary outcomes

Muscle strength is being assessed by a hydraulic hand dynamometer (JAMAR, SAEHAN corporation, Changwon, S. Korea), and isometric mid-thigh pull (Baseline leg dynamometer, Fabrication Enterprises Inc., White Plains, NY, USA) and cardiovascular fitness (predicted maximal oxygen uptake, VO_2max_) is being assessed by a sub-maximal exercise test on a cycle ergometer (Monark 928E, Monark Exercise AB, Vansbro, Sweden). Pressure pain thresholds are assessed using a pressure algometer (Somedic Sales AB, Hörby, Sweden). Blood sampling and muscle biopsy procedures are taking place at the Clinical Trial Unit laboratory at Radiumhemmet, Karolinska University Hospital, Stockholm. Muscle biopsies (approximately 100 mg) will be obtained under local anesthesia from the vastus lateralis muscle (lateral part of the knee extensor muscle) with a Bergström needle [28]. This takes place before the intervention starts and 24–72 h after the last training session. Contractile function, markers for systemic inflammation, muscle fiber area, mitochondrial density, and expression of genes important for muscle adaptation will be analysed. Concurrently, venous blood is sampled for markers of systemic inflammation, hemoglobin levels, genotyping, and for analysis of white cell distribution and epigenetic patterns.

Patient-reported outcomes include self-reported health-related QoL assessed by the European Organization for Research and Treatment of Cancer (EORTC QLQ-C30) questionnaire which is a cancer-specific instrument that measures health-related QoL in terms of physical, emotional, social, cognitive and everyday function. Common cancer-related symptoms are assessed by the Memorial Symptom Assessment Scale (MSAS) [29]. The instrument has been validated for Swedish patients with breast cancer [30].

### Additional measures

Additional measures include height, body mass, exercise session attendance and adherence, haematological parameters, cancer treatment, recurrence, survival data, sick leave data, co-morbidities, and cause of death in relevant registers. Sense of coherence (SOC) will also be assessed by a questionnaire (SOC short version which consists of 13 questions) that all participants complete once at baseline testing [31]. The Swedish version of the SOC-13 has been shown to have good validity and reliability. It has also demonstrated high internal consistency [32]. Participants are being asked to wear an accelerometer (ActiGraph model GT3X+, ActiGraph LCC, Pensacola, FL, USA) on an elastic belt over the right hip during all waking hours, except during water based activities for seven consecutive days directly after baseline testing, at one, two and five years after the initial 16-week intervention to objectively record daily activity (sedentary behaviour and physical activity) patterns. Data will be analysed using standardised cutoff points and valid wear-time criteria [33]. Participants will also be asked to complete an Activity Diary to assess daily subjective physical activity during the 16-week intervention period.

### Data entry, management and analysis

Data is collected and entered exclusively by the study research staff. All hard copies of forms are kept in a locked filing cabinet at Karolinska Institutet. Data are entered into computer files in a re-identifiable format (participant numbers only) and password protected. Participants will not be identified in the resulting manuscripts and reports. The data monitoring committee for the current study is made up of the research project staff only and does not include representatives from the funding bodies, nor will it be influenced in any way by these funding bodies. Only the study researchers have access to the dataset.

### Confidentiality

Information collected directly from participants will be in re-identifiable form and any information collected for, used in or generated by this project will not be used for any other purpose. Only the study investigators will have access to study information.

## Intervention

### Phase I: Initial intervention- Supervised exercise training

Participants in the exercise groups begin training the week after the initial testing session which takes place prior to the patients’ second session of chemotherapy. Both exercise groups train twice per week on non-consecutive week days for 16 weeks. Each session is approximately 60 min in duration and is conducted at the exercise clinic at the Karolinska University Hospital. An exercise physiologist or oncology nurse supervises all sessions to ensure safety, correct technique and adherence to the exercise protocols. Participants can choose to perform the continuous aerobic exercise on a cycle ergometer, elliptical ergometer, or treadmill, the interval aerobic exercise is performed on a cycle ergometer. Resistance exercises are performed using machine resistance training equipment, participants’ body weight and free weight dumbbells and barbells. All exercise sessions commence with a five min warm up on a cycle ergometer, treadmill, or rowing machine at a rating of perceived exertion (RPE) of 10–12 on the Borg scale [34] and sessions conclude with a 10 min cool-down which includes dynamic and static muscle stretching activities of the major muscle groups.

Group 1 (AT) exercise sessions commence with 20 min of moderate intensity continuous aerobic exercise at an RPE of 13–15. This is followed by 3 × 3 min bouts of high intensity intermittent aerobic exercise at an RPE of 16–18 interspersed with ~1 min of passive or active recovery.

Group 2 (CT) completes both resistance exercises and high intensity intermittent aerobic exercise during each session. The resistance training regimen consists of 8 exercises (leg press, biceps curls, triceps extensions, bench press, shoulder press, standing row, sit ups/Russian weighted abdominal twist, and prone lying back extensions. Participants complete 2 sets of 8–12 repetitions at an initial intensity of 70% of their estimated 1 repetition maximum (1-RM) strength, and increase to 80% of estimated 1-RM when more than 12 repetitions can be correctly performed by the participant. Every four weeks estimated 1-RM strength tests are performed to ensure progression. Group 2 also completes 3 × 3 min bouts of high intensity intermittent aerobic exercise (RPE = 16–18) interspersed with ~1 min of passive or active recovery.

Group 3 (UC) serves as the control group and receive usual care for breast cancer and general information on physical activity but no supervised exercise training or prescription.

### Phase II: Follow up - Support to maintain physical activity after the intervention

Physical activity on prescription (PAP) is a personalised written prescription of physical activity [35] that will be used to support the women participating in the two exercise arms to maintain their physical activity levels throughout the entire duration of phase II of the study. Participants will have the opportunity to train at the gymnasium operator Friskis & Svettis at locations throughout Stockholm. Motivational seminars on a healthy lifestyle, fitness, and training options will be held in collaboration with Friskis & Svettis three times a year at their facility in Hagastaden, Stockholm. The research team will be responsible for providing the motivational seminars and physical activity prescriptions.

## Statistical analyses

Data will be analysed using the IBM SPSS (Statistical Package for the Social Sciences) 20 statistical package for Windows (SPSS, Chicago, IL, USA). Analyses will include standard descriptive statistics (one-way ANOVAs or Kruskall-Wallis tests, as appropriate, and Chi squared tests). ANCOVAs accounting for baseline values will be used for the main analysis. Variables with data that are not normally-distributed will be log transformed prior to ANCOVA analyses. Paired t-tests or Wilcoxon signed rank tests, as appropriate, will be used to test for within groups differences from baseline to follow up. All tests will be 2-tailed and statistical significance set at *p* ≤ 0.05. An intention-to-treat approach will be used for the analyses with missing values replaced using the expectation-maximization method.

Power Calculation: With fatigue as the primary outcome measure, a sample size of 65 patients/group is required, based on an effect size of 0.53 and power = 0.8. From our research group’s experience with participant dropout in previous exercise trials in cancer survivors, we accounted for an attrition rate of ~20% and therefore are aiming to recruit 80 participants into each group.

## Monitoring harms

### Adverse events

Study supervisors record any adverse events that occur as a result of the testing or training activities of the study. Participants are also asked to directly report any adverse events they experience. Reported adverse events are recorded by the research staff and reported both to the relevant ethics committee and as relevant in any subsequent publications and reports. Patients who experience an adverse event will be referred to the relevant health professional for a medical assessment. All participants will be covered by the Patient Insurance that is made available for participants in clinical studies at Karolinska University Hospital.

### Additional potential discomfort issues

Progressive resistance training and muscle strength testing are known to result in mild discomfort, delayed onset muscle soreness and joint stiffness especially in untrained individuals. However, participants are informed of the possibility of delayed onset muscle soreness, symptoms and procedures to alleviate them. Warm-ups and cool-downs are incorporated in each testing session to mitigate muscle soreness and return the individual to their resting heart rate level before leaving the training facility. There is also the risk of a muscle strain. However, this risk is reduced by careful monitoring of each participant, ensuring that correct loads are used, checking technique and requiring a warm-up and cool-down period with each exercise session. Furthermore, all testing sessions are conducted with one participant at a time, and exercise training takes place in small groups of up to five participants, each session supervised by the study exercise physiologist or oncology nurse.

There may be a low risk of falling when performing the functional tasks; however, participants are closely supervised. There may also be some discomfort from the procedures involved in blood samples and muscle biopsies and some bruising may result. However, both procedures are performed by a trained phlebotomist (blood sample) and an experienced physician (muscle biopsy).

## Discussion

This study will provide a more complete understanding of the effects of different modalities of exercise on the physical and mental health of breast cancer survivors in the short and long term as well as adding further knowledge regarding the potential exercise may have in managing fatigue, the most commonly reported but poorly understood side-effect of cancer diagnosis and treatment.

There is a concern by some health professionals and researchers, both within and outside of the oncology setting, that high intensity exercise training may not be feasible or safe for individuals with a history of cancer. However, recent controlled trials have adopted high intensity training and showed exercise induced physical adaptations to be superior to low or moderate intensity exercise [18, 36] and concluded that this mode of exercise to be safe and well tolerated in both patients undergoing chemotherapy [18, 37] and cancer survivors [38–40].

The results of the study will be communicated to all participants at the conclusion of the trial. The findings of the study will be shared with the public and research community by way of scientific peer-reviewed publications and presentations at scientific meetings. Popular science articles will be written to inform the public of the study results and study findings will be communicated to the relevant organisations and funding bodies for dissemination to relevant health care and policy professionals.

In terms of advancement of cancer survivor care, we expect that dissemination of the knowledge gained from this study to contribute to developing effective strategies to improve the physical and psychosocial health of breast cancer survivors. In particular, we expect that the knowledge will aid our understanding of the benefits and issues associated with long term exercise programs.

This randomised controlled trial will generate substantial information regarding the effects of different types of exercise on the health of patients with breast cancer undergoing chemotherapy. We anticipate that the results of this study will help to develop future exercise programs and interventions that are more effective and appropriate for and attractive to patients with breast cancer.

## Abbreviations

AT: Aerobic exercise training
BMI: Body mass index
CT: Combined resistance and aerobic exercise training
EORTC QLQ-C30: European Organization for Research and Treatment of Cancer Core Quality of Life questionnaire
MSAS: Memorial Symptom Assessment Scale
PAP: Physical activity on prescription
QoL: Quality of life
RM: Repetition maximum
RPE: Rate of perceived exertion
SOC: Sense of coherence
UC: Usual care control group
VO2max: Maximal oxygen uptake
